# V-CarE—A Conceptual Design Model for Providing COVID-19 Pandemic Awareness: Proposal for a Virtual Reality Design Approach to Facilitate People With Persistent Postural-Perceptual Dizziness

**DOI:** 10.2196/38369

**Published:** 2023-07-27

**Authors:** Syed Fawad M Zaidi, Niusha Shafiabady, Shereen Afifi, Justin Beilby

**Affiliations:** 1 Department of Business Information Systems Torrens University Australia Sydney Australia; 2 Faculty of Science and Technology Charles Darwin University Sydney Australia; 3 Faculty of Media Engineering and Technology German University in Cairo Cairo Egypt; 4 Torrens University Australia Adelaide Australia

**Keywords:** COVID-19, immersion, pandemic, Persistent Postural–Perceptual Dizziness, PPPD, virtual reality

## Abstract

**Background:**

Virtual reality (VR) technology has been solidifying its ground since its existence, where engagement and a sense of presence are key. The contemporary field of development has captured the attention of researchers due to its flexibility and compatibility attributes. During the COVID-19 pandemic, several research outputs have shown promising prospects of continuing research in the field of VR design and development—in health sciences including learning and training.

**Objective:**

In this paper, we aim to propose a conceptual development model named V-CarE (Virtual Care Experience) that can facilitate the understanding of pandemics when it comes to a crisis, taking precautionary measures where needed, and getting used to certain actions for preventing pandemic spread through habituation. Moreover, this conceptual model is useful to expand the development strategy to incorporate different types of users and technological aid as per need and requirement.

**Methods:**

For a detailed understanding of the proposed model, we have developed a novel design strategy to bring awareness to the user about the current COVID-19 pandemic. VR research in health sciences has shown that with appropriate management and development, VR technology can efficiently support people with health issues and special needs, which motivated our attempts to explore the possibility of employing our proposed model to treat Persistent Postural-Perceptual Dizziness (PPPD)—a persistent nonvertiginous dizziness that could last for 3 months or more. The purpose of including patients with PPPD is to get them engaged in the learning experience and to make them comfortable with VR. We believe this confidence and habituation would help them get engaged with VR for treatment (dizziness alleviation) while practicing the preventive measures during the pandemic in an interactive environment without actually facing any pandemic directly. Subsequently, for advanced development using the V-CarE model, we have briefly discussed that even contemporary technology like internet of things (IoT) for handling devices, can be incorporated without disrupting the complete 3D-immersive experience.

**Results:**

In our discussion, we have shown that the proposed model represents a significant step toward the accessibility of VR technology by creating a pathway toward awareness of pandemics and, also, an effective care strategy for PPPD people. Moreover, by introducing advanced technology, we will only further enhance the development for wider accessibility of VR technology while keeping the core purpose of the development intact.

**Conclusions:**

V-CarE–based developed VR projects are designed with all the core elements of health sciences, technology, and training making it accessible and engaging for the users and improving their lifestyle by safely experiencing the unknown. We suggest that with further design-based research, the proposed V-CarE model has the potential to be a valuable tool connecting different fields to wider communities.

## Introduction

Recently, virtual reality (VR) technology has received growing attention in diverse areas and a plethora of research. VR creates an immersive 3D visual experience and sensory environment trying to mimic the real world using simple wearable devices. Interestingly, VR technology has been widely used as a cost-effective tool in advanced fields of education, health, engineering, and medicine. Immersion, presence, and interactivity are key factors in VR [1-5]. We believe that these factors mark the prominent capability of VR, which has drawn great attention in the field of education, learning, and health sciences [6-11]. VR has significantly improved the education and skills of health professionals [10,12], but further research is still required to study the effectiveness of more immersive and interactive forms of VR in different settings. In this study, we have proposed a VR-based model that incorporates education and health science elements to examine and evaluate the effectiveness of a 3D immersive experience in learning and training.

**Figure 1:**
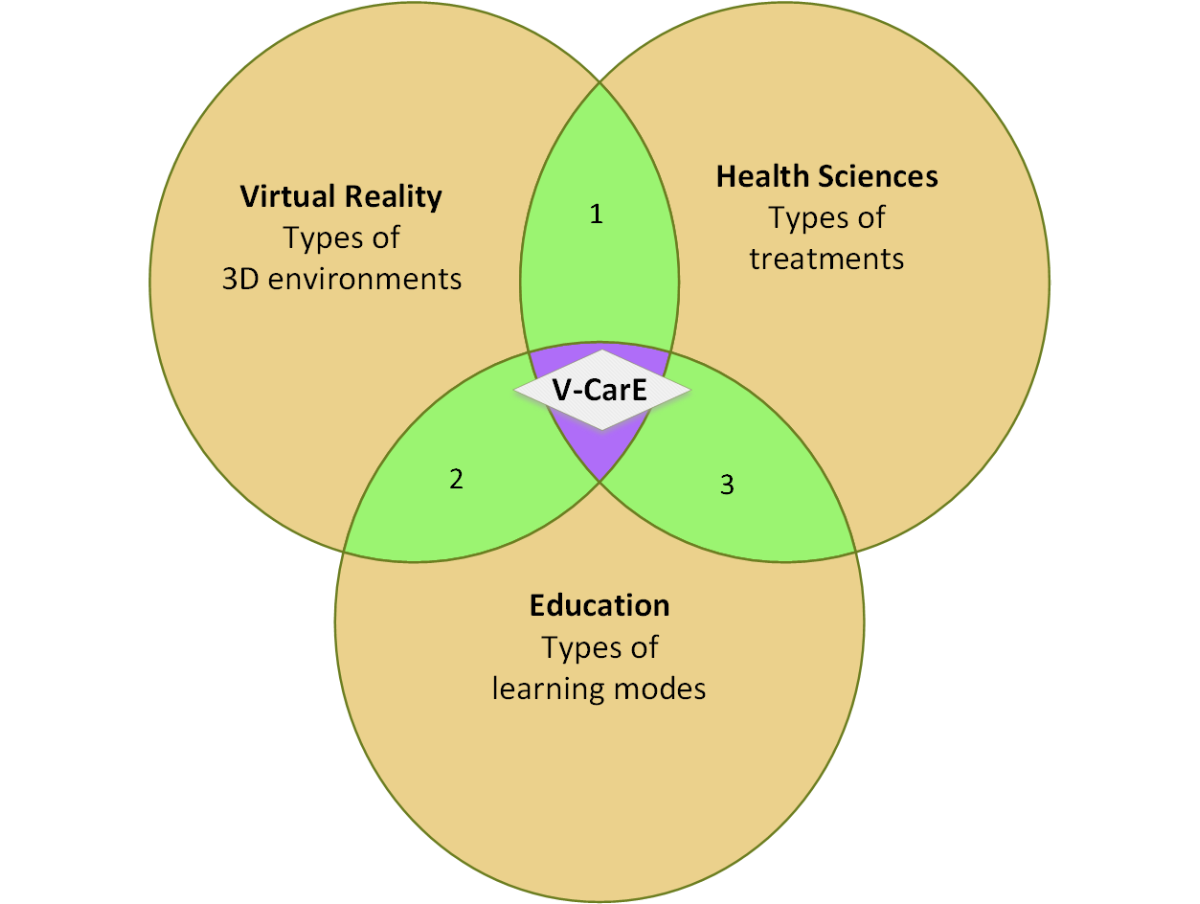
V-CarE (Virtual Care Experience) conceptual model.

The proposed VR conceptual framework, V-CarE (Virtual Care Experience) model, can be applied by VR developers to create a 3D simulation incorporating educational and health elements (Figure 1). The desired 3D environment would give the users an immersive experience to practice how to react in a serious situation, which would eventually benefit their health through their actions. The proposed model is flexible and can be generalized for different purposes, including engagement and habituation. This would cater for a wider acceptance of the technology, which will contribute to public health and serve the community. It is worth noting that the internet of things (IoT) would be incorporated within our proposed model for providing a 3D and fully immersive simulation. The users would experience real situations developed objectively with required surroundings and settings for different training cases and purposes. IoT is a system that connects different objects or things in one network that has been widely used in numerous applications in a wide range of domains like health care, agriculture, and smart homes [13-15].

Furthermore, the V-CarE model aims to enhance awareness of pandemics during unforeseen circumstances, implement necessary preventive measures, and encourage habitual actions for disease prevention. The COVID-19 respiratory illness outbreak started in China in 2019 [16]. It spread all over the world, resulting in a global pandemic in less than a year [17]. In an attempt to control its spread, preventive measures, and social distancing were suggested based on the situation and conditions of the outbreak [18].

With the rise of the prevalence of COVID-19, VR played an important role in fighting the pandemic by providing a virtual and safe environment for leveraging remote interactions and connecting people. It can be used to change individual behavior while maintaining social distancing through immersive learning and training through 3D simulation. A web-based survey was introduced to study the importance of using VR to help people improve their mental health and physical well-being during the lockdown [19]. Many VR-based apps have been recently introduced in the context of the COVID-19 pandemic to help in cognitive and physical rehabilitation therapies, web-based education, training, and treatments [20-23]. However, there is no strategy for implementing and practicing preventive measures such as social distancing to accommodate performing normal work procedures in the industry, education, health care, and other fields. The proposed V-CarE model is developed to educate people about COVID-19 through VR immersive environment experience, focusing on implementing preventive measures, and social distancing strategies for controlling the disease spread.

To test and validate the extendible capabilities of V-CarE, we have applied our proposed model to people with Persistent Postural–Perceptual Dizziness (PPPD). V-CarE model has been designed with strategies for preventive measures to avoid triggering the symptoms. PPPD has been identified as a vestibular disorder that causes persistent dizziness and unsteadiness for 3 months or more [24]. Other than this study, very limited research has been done on using VR simulation as a cost-efficient tool for understanding and managing patients with PPPD through exposure therapy [25-28]. It is very challenging for patients with PPPD to follow the preventive measures that are crucial nowadays to control the current COVID-19 pandemic. Recently, there has been some significant work conducted on the application of VR and its capabilities to handle situations during the COVID-19 pandemic. Telemedicine services, mental health support, remote work collaborations, and similar engaging activities have been potential areas of research in VR development. Engagement and interactive rehabilitation experiences have been the main focus of the research as COVID-19 largely impacted daily life activities and approaches toward looking at day-to-day activities for the future [29-32]. Despite progress, there has been no discussion on how VR development strategies are made to keep the elements of entertainment, engagement, and interactivity intact for the wider audiences. In this study, we have highlighted the need for a proper visual map and introduced a conceptual model that can involve experts in their fields to connect strongly and develop an effective 3D simulation addressing the needs of the users. Moreover, we have also suggested that V-CarE, being a promising development model, can also facilitate people with PPPD who are already going through the complexity of living that comes with the symptoms [33].

Therefore, this study aims to employ the proposed V-CarE conceptual model for VR developers to provide people with awareness of the pandemic and its preventive measures through 3D immersion. Also, we have highlighted that the similar 3D simulation developed here can facilitate people with PPPD. The product can further be enhanced with little to no major change, whereas contemporary technology (eg, IoT) can be incorporated into the V-CarE–based design for a wider accessibility. Finally, we have concluded that the V-CarE approach positively impacts daily life at a time when the pandemic crisis and personal dizziness struggles are the major health issues.

## Methods

### The Proposed V-CarE Model

The term V-CarE is inspired by the term “We Care,” which stands for Virtual Care Experience, which refers to the concept that VR technology positively impacts people’s lives. People would learn and train themselves to remain safe and keep themselves healthy using this technology. V-CarE is designed to be a general and flexible learning and training model that provides an immersive 3D experience for the users to have a healthy lifestyle through improved habits and daily activities. It can also help in understanding and building awareness of different pandemics, as well as practicing its required preventative measures through habituation. In addition, V-CarE represents a conceptual framework that facilitates further refining the investigation process of understanding primary symptoms among patients (users) for researchers and clinicians. In addition, it can assist researchers to handle cross-disciplinary work more objectively.

**Figure 2:**
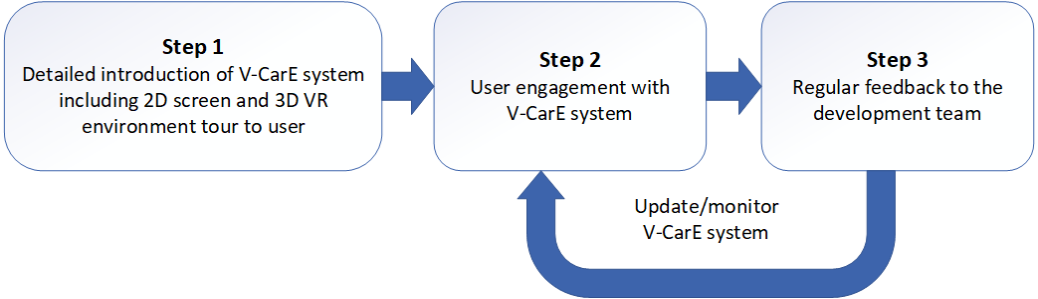
V-CarE (Virtual Care Experience) standard engagement protocol. VR: virtual reality.

As the V-CarE concept resides at the core of 3 different major fields, including VR (3D environment), education, and health sciences, it is important to go through each field and reach the core (V-CarE) design as shown in Figure 1. In this way, the design can be easily customized as per the needs of the development. Table 1 describes how the 3 generic areas are set together and blended into 1 core that would have the essence of all the major fields. Based on the required type of VR environment, the order of considering mapping of sets would vary in each row of Table 1, but it would remain in the same column sequences (from left to right). To develop the right product at the end, the V-CarE team are expected to have experts in the 3 fields (education, VR, and health sciences), so that all common elements of the 3 major fields are addressed accurately. Once the V-CarE–based product is ready, to continue keeping the positive impact of the product based on the V-CarE concept, we have proposed a standard protocol as depicted in Figure 2 to make users feel comfortable with the VR experience.

**Table 1:** Descriptive table for the proposed V-CarE conceptual model in Figure 1.

V-CarE–the 3 sets and common elements		
Types	Venn diagram number–types	V-CarE model components
Virtual reality (VR)–3D environments (commercial entertainment, educational, medical, manufacturing, therapy, mixed reality, etc)	1. Virtual reality and health sciences: medical VR, VR therapy.	VR-based experience model to improve user and patient’s symptoms, learning, and awareness affecting virtual rehabilitation therapy.
Education–modes (learning and training; conventional classrooms, web-based environment, virtual environment, blended environment, etc)	2. Virtual reality and education: educational virtual environment	VR-based experience model to improve user and patient’s symptoms, learning, and awareness affecting virtual rehabilitation therapy.
Health sciences–treatments (medicine therapy, cognitive behavioral therapy, vestibular rehabilitation therapy, etc)	3. Health sciences and education: patient education in general (learning about therapies and their effectiveness)	VR-based experience model to improve user and patient’s symptoms, learning, and awareness affecting virtual rehabilitation therapy.

### V-CarE Model’s Design and Development Framework

As mentioned earlier, V-CarE is a conceptual model that can be used with flexibility for different situations as long as it is linked with training, learning, and maintaining good health (Table 1). The V-CarE team would involve individuals from areas of health sciences, VR, and education.

### The Protocol of Introducing V-CarE to Users

A standard protocol has been laid out to introduce the V-CarE system, aiming to make users comfortable with the VR experience based on the V-CarE concept. The user would go through the proposed standard process when using V-CarE as described in Figure 2. They would be introduced to the system thoroughly, including the VR experience at first. Then, the product will be used by the users at home at their convenience. With every growing demand and experience requirement, the system would continuously improve through feedback where necessary to give the user an ultimate experience of a VR environment.

**Figure 3:**
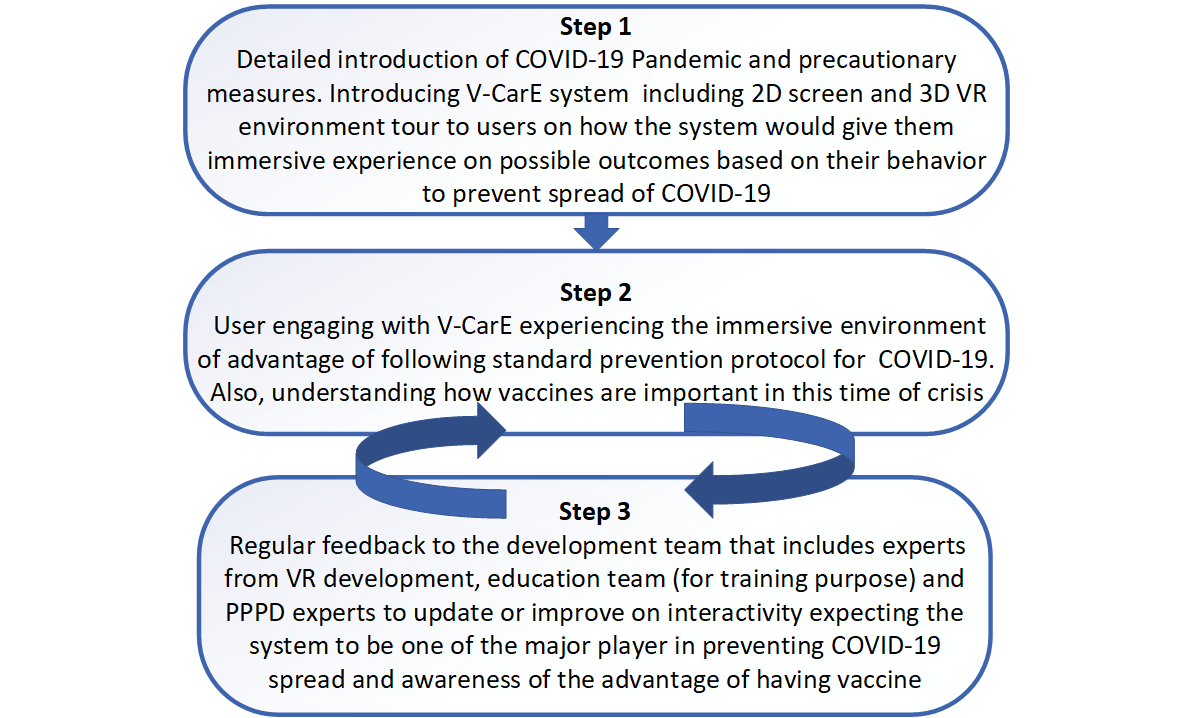
The protocol for using V-CarE (Virtual Care Experience) system for COVID-19 pandemic awareness. PPPD: Persistent Postural-Perceptual Dizziness; VR: virtual reality.

In this case, we will use the proposed general process of using the V-CarE system for COVID-19 pandemic awareness. Figure 3 shows the updated protocol that was designed toward introducing an immersive experience dedicated to providing an understanding and awareness of the current pandemic, as well as facilitating a pathway for the user to get used to the required preventive measures and the vaccination process.

### Design Strategy—Educating to Combat (COVID-19) Pandemic

In this study, looking at the seriousness of the COVID-19 pandemic, we have proposed a virtual learning environment design using the V-CarE model that would help people to understand the pandemic, educate themselves, and take necessary measures consistently in a hassle-free manner. The 3 design steps for implementing the VR immersive strategy and experience are as follows:

1. From the 3 broad sets (highlighted in yellow color in Figure 1)—the V-CarE design requires understanding the elements to choose for subsets to develop a relevant VR environment. For our purpose, our final set should have these 3 components: a VR environment for an interactive experience, education about COVID-19, and exposure therapy to impact the users’ lives positively.
2. From the 3 blended sets (highlighted in green color in Figure 1)—the V-CarE design requires a VR environment with an educational aspect in it. Education about what measures can reduce the chances of getting infected with COVID-19, its symptoms, information on SARS-CoV-2, and the 3D immersive experience of the measures to keep oneself safe.
3. From the core V-CarE set (highlighted in gray)—The V-CarE design requires a VR environment where users can experience the results of preventive measures at home during the lockdown through a 3D simulation. The virtual simulated environment is designed to be an indoor environment; however, it can be developed to be outdoors when required. In the 3D-immersion experience, users can safely interact with the VR environment and perceive a deep awareness of the COVID-19 virus.

### Development Strategy—3D-Simulated Experience

In this section, we provide an example of developing strategies through a simulated environment for users to practice key preventive measures and social distancing procedures. The virtual simulation will have the following different scenes in a house:

1. Scene in the bathroom where a user must wash hands thoroughly for 20 seconds (timer is active). If any part of the hand is left unattended, a red spot will be added to the simulation environment. This spot would grow if the user does not attempt to wash hands again for 20 seconds.
2. Scene in the house where there is a grocery item that is located on the table. The red spots on the item would be an indication of the possible risk of COVID-19. A wipe, and a water tap in the kitchen would be available. The user would be required to clean the item with a suitable option.
3. Someone knocks at the door and the user would be asked to take action; (1) open the door, and (2) ask the person to keep a 2-meter distance (social distancing) from the door before interacting.
4. Social distancing scene where green circles depict moving users if they are moving or standing at 2-meter distances or more. If the distance becomes smaller, the circle’s color will turn red accompanied by a warning alarm.
5. To keep the immersion engaging, these proposed scenarios would undergo a slight change with each experience, which can be decided by the developers. Also, these scenarios can be easily adapted or redesigned to address different procedures and precautions (matching the rules of the lockdown’s level). Consequently, more scenes can be added to address more of the suggested and updated preventive measures for indoors and outdoors, aiming for a complete training and therapy system. Moreover, a scene can be added for understanding and getting familiar with the special polymerase chain reaction (PCR) test used to diagnose COVID-19 and for the vaccination process. With VR immersion, telepresence, and real-time interactions, people would get more engaged and motivated to use VR technology to increase their awareness about COVID-19 and practice preventive measures instead of just reading and hearing about the disease through media. User-centered tutorials and demonstrations would be used to assist the users in using the system independently and would provide further support to the users [34].

### Sample Simulation Process Using V-CarE–Visual Aid

**Figure 4:**
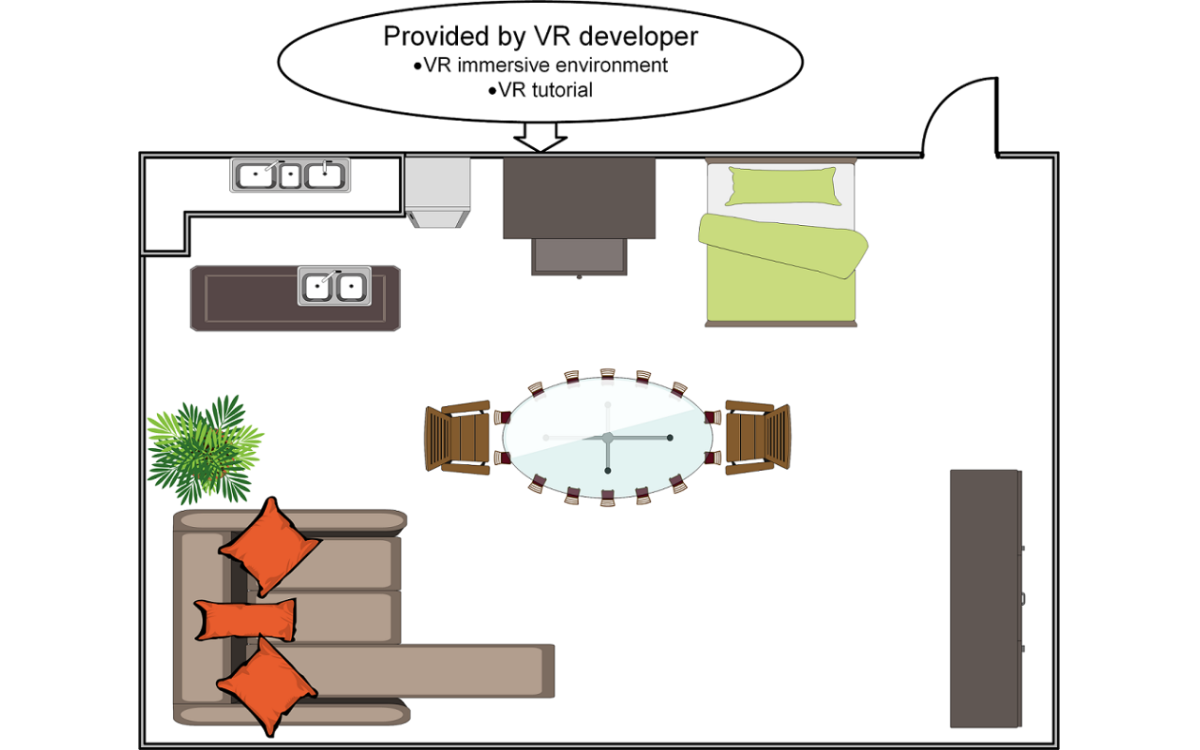
Design layout I–studio room. VR: virtual reality.

For a visual understanding of V-CarE, we have provided an example of a studio room (in 2D for simplification purposes): the VR version can be customized as needed. Users would go into a 3D simulation of the room. In a conventional VR development environment, understanding interactions with objects in the room will be decided by the VR developers, whereas in the V-Care framework, these interactions will be guided by expert judgement through guided tutorials. It is expected that the interactions in the room can either be a part of a VR-based game or solely a VR experience (Figure 4).

**Figure 5:**
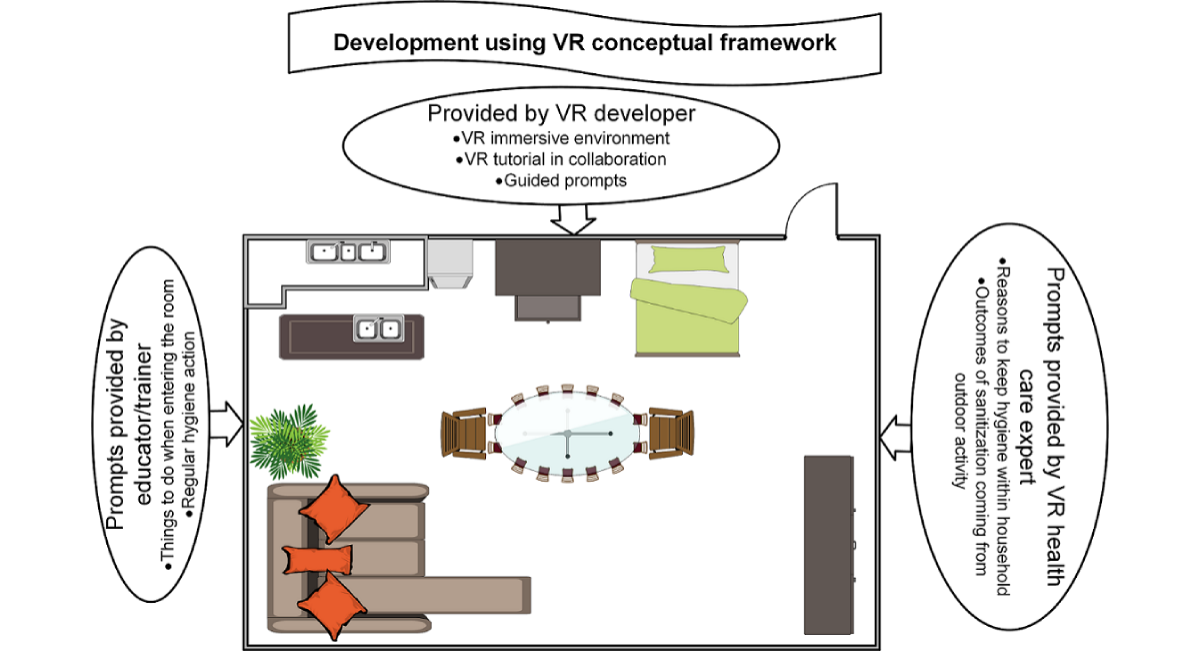
Design layout II–studio room from V-CarE perspective. VR: virtual reality.

If the environment is developed using the V-CarE framework, the elements in the VR experience would have attributes infused by experts from different disciplines and the purpose would be narrowed down to specific yet common goals. In this study, the focus would be to understand the actions required to avoid COVID-19. As mentioned before, experts from the 3 disciplines would be involved in the V-CarE–based product development. The user would (1) interact with the environment (VR developer would be involved), (2) receive continuous input feedback (educators and trainers would be involved) to be informed of the necessary steps to avoid COVID-19, and (3) get prompts of health information on the actions they took in the immersion (Figure 5).

As the virtual environment is flexible to the users’ needs, the idea of a descriptive VR environment is left to the developers. Figure 5 illustrates VR environments that can be created from different perspectives. At the same time as design layout II in Figure 5 shows, a simple environment like a studio room can be used as an insightful interactive space to tackle the spread of COVID-19 through community contribution and awareness of the pandemic. Some of the recent research work showed that the developers have started investigating strategies around creating casual VR simulations for certain groups of audiences [35-38]. In this study, we have proposed a conceptual framework to engage experts from other disciplines to keep the scope of VR simulation balanced to the needs of the users. This scope can easily be expanded to the direction of selected disciplines without compromising on the quality of the developed VR product.

### Incorporating Internet of Things into V-CarE Design Strategies

The development of our proposed V-CarE model will require the involvement of people from 3 different fields of expertise including education, VR, and health. It is important to see how V-CarE can collect the users’ feedback and reaction to connect them to the right resources. The IoT has been around for some time and the promising attribute is the integration and communication of several technologies [13], and it fits well with the proposed conceptual V-CarE model.

In the integration of VR and IoT, Empathic VR is an appropriate example that can support the advantages of V-CarE for its flexibility in adopting IoT. Empathic VR uses a VR head-mounted display with a mobile remote–controlled robot. This enables 2 people at distant locations to connect and move freely within a real world and virtual workspace while creating a strong sense of telepresence of actually being in the same location [39]. In the proposed V-CarE model, an IoT-based system can be designed to suit the users’ needs by involving the relevant health experts to facilitate a deeper understanding of the user experience and to support users in line with health care practices.

**Figure 6:**
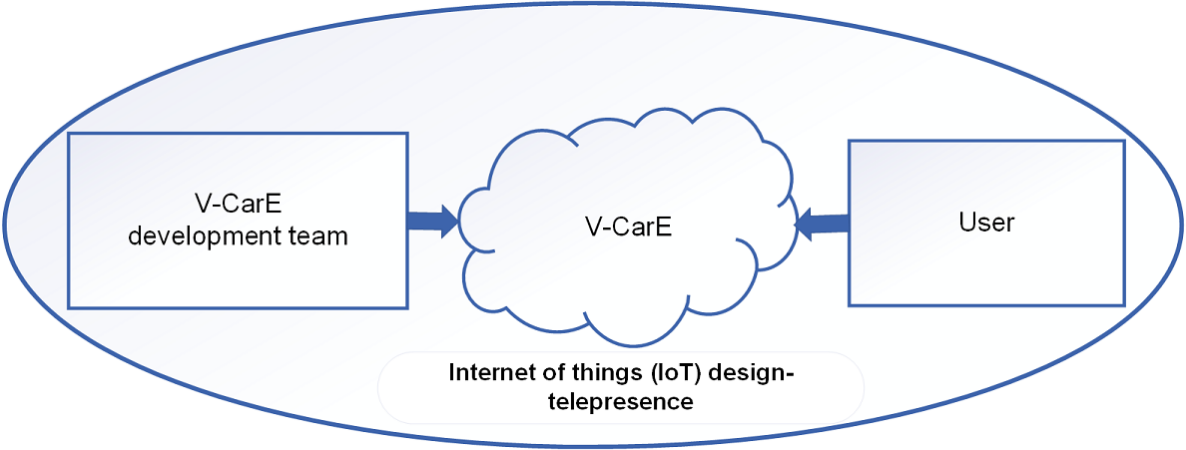
V-CarE (Virtual Care Experience)–proposed framework for IoT compatibility with the V-CarE model.

Figure 6 depicts our proposed framework that engages both the developers and users using the V-CarE model, aiming for an efficient and dedicated IoT-based VR immersive engagement. As for COVID-19, it is evident that social distancing and isolation are necessary to alleviate the spread of the virus, and IoT-based design would be ideal to connect everyone involved in helping the society become COVID-19-free. Interestingly, modern IOT and internet of medical things technologies have been studied to assist in managing the current pandemic [35]. Therefore, our proposed model will be empowered by incorporating these promising technologies, aiming to achieve an efficient system with a variety of applications including managing the COVID-19 pandemic.

### Extending V-CarE for Patients With PPPD

With V-CarE, users can immerse and experience reality through 3D simulation and learn according to their engagement preferences. The Venn diagram–based model (Figure 1) can adapt to changes and various considerations as long as the core V-CarE has all the common elements from the 3 big sets (VR, education, and health sciences). Also, we would like to stress that the V-CarE model can help people with different disorders, symptoms, and phobias. In this case, we would like to see how people with PPPD can benefit from the current setup. PPPPD is an inconsistent chronic vestibular nonvertiginous form of dizziness and unsteadiness that persists for 3 months or more [24]. It exacerbates by upright position, passive and active motion of oneself, and exposure to static and dynamic complex environments which are overwhelming to visual perception. Having entered the list of the International Classification of Disorders-11 in 2015, there are very limited research studies on the treatments and effects of PPPD and the impact on the affected people [36]. Recently, VR technology has been exploited for vestibular rehabilitation through balance and habituation exercises [37]. There are very limited studies, including this work, on using VR to understand PPPD through exposure therapy [25-28]. None of the studies discussed the encouragement and trust factors of VR technology.

Considering the triggers of PPPD and its symptoms, using VR would be a promising starting point for patients with PPPD to get used to the environments and to understand and practice immersion [28,40]. They would be able to see and feel the environment of a house where their daily activities would be similar to what they are expected to do, especially during the COVID-19 pandemic. The proposed V-CarE model with the designed strategy for COVID-19 will provide the required immersion and sense of achievement for PPPD users to practice following all preventive measures. This will make them engaged while experiencing the outcome of their actions in the real world. Not only would they have a virtual experience of learning, but they might also get comfortable with virtual headsets through habituation. This will lead to their acceptance of VR technology and, later, using it by choice for their treatment (self-therapy) under the supervision or advice of PPPD experts.

Moreover, if the simulation is similar to their home environment (that does not trigger dizziness symptoms), people with PPPD would interact comfortably, reducing fear responses. Furthermore, for the sake of knowing COVID-19 and doing their part to control the spread, they would take initiative in practicing COVID-19 preventive measures through 3D immersion. The proposed protocol introduced in Figure 3 will help in creating awareness for patients with PPPD and assisting them to overcome the symptoms. The PPPD experts would be involved in the development process of the system and introduce it efficiently to patients with PPPD. In addition, incorporating IoT into the proposed model would offer a more relaxed environment with familiar surroundings, aiming to improve habituation and adaptation. Additionally, the proposed model would help people with PPPD to understand the PCR test, as well as the vaccination process, which would assist in controlling the COVID-19 spread.

The proposed model presents a simple and feasible VR-based solution that can be applied to help patients with PPPD (besides regular users) understand the COVID-19 pandemic and practice the needed precautions and preventive measures.

### Electronic Equipment for V-CarE Design and Development

VR has come a long way to get the attention of potential users and create a competitive market, with various companies putting in effort to keep their products as one of the leading equipment in the market [41,42]. HTC Vive and Oculus are keeping their gadgets accessible for consumers and developers with several models and compatible software design kit. The latest graphics cards combined with powerful machines have been the major contributors to the VR success, considering the framerates, and close to real-world development of simulation. Augmented reality has even gone one step further to cater for the necessary engagement and interactive factor which VR is providing to the users [43]. V-CarE can be applied to any design methodology using the hardware in line with the users’ and participants’ needs.

### Ethical Considerations

The paper proposes a theoretical design conceptual model without involving any human subjects. The design element focuses on the possible development of VR interventions that can sustain positive behavior and mitigate the negative effects of the pandemic and PPPD symptoms on daily life activities.

### Usability Testing

To materialize the conceptual framework, we suggest rigorous usability and user testing to make the product reliable and effective for end users. Experts in the areas of VR, health sciences, and education, can collaborate in cross*-*disciplinary domains to harvest quality outcomes using VR technology [10,12,27,44]. The proposed V*-*CarE framework suggests that for the development phase, the relevant stakeholders would be involved in testing the system’s usability. The process should engage experts in their fields and select users from a group of people who would be directly impacted. It is important to involve the people at the right time for the right role in the development of the VR environment [34,45]. Tools like System Usability Scale and me CUE have been extensively used by researchers and practitioners to measure usability and user experience [46,47]. Furthermore, the agile project management approach and scrum methodology [40] would be appropriate methods to move to the development phase of the V*-*CarE model, considering its nature of involving researchers from different fields.

### Sample Size

Although the study primarily focuses on users’ COVID-19 *a*wareness, we have also emphasized that the design has the potential to cater to a broader audience, including individuals with PPPD. Preliminary research and investigation have been performed as proof of concept for using VR technology to help patients with PPPD in their journey of exposure therapy and improving symptoms. For example, in recent studies, a VR prototype has been proposed*,* paving the way toward an efficient VR-based intervention tool for improving exposure therapy *through* immersion strategy [27]. In addition, a smart VR-based e-diary is proposed for improving PPPD therapy and facilitating habituation [28]. For appropriate sample size calculation, priori analyses were performed to estimate a suitable sample size for a reasonable power level. The G*Power (version 3.1; Universität Düsseldorf) statistical program was used for sample size calculation and power analysis [49*,*50]. It was calculated that 54 participants can designate 95% power of the study based on a medium effect size Cohen *d*=0.5, with significance level α=.05 (*2*-tailed *t* test). Therefore, we suggest including a total of 54 participants *and* patients (assuming some dropout), aiming to achieve 95% statistical power of the study. The sample of participants can include both genders (male *and* female). For gathering information and collecting feedback for the experiment, an efficient questionnaire *or* survey is required based on the standards for developing questionnaires for educational research and using the handbook of questionnaire development as a guide [50].

## Results

We have proposed a model that provides a significant step toward enhancing the accessibility of VR technology. The methods section highlights that VR development using V-CarE can strengthen public awareness regarding pandemics. Also, VR development can provide effective care to individuals with PPPD without making major VR exposure training improvements. In addition, we have determined that incorporating cutting-edge technology, for example, IoT, into the model will facilitate the expansion of VR accessibility while preserving the primary purpose of the development.

## Discussion

### Principal Findings

The V-CarE conceptual model is a major leap forward in the strategic use of VR technology for health care; and educational purposes–at a user level. By developing a fully immersive, semi-immersive, or nonimmersive 3D simulation, researchers from the fields of education, health, and creative technology can work together to improve health care outcomes and address the challenges posed by the COVID-19 pandemic.

The model provides a pathway to develop a virtual experience that helps people understand COVID-19 and the recommended preventive actions. In addition, the model has the potential to be applied in a wide range of settings, from medical clinics and hospitals to educational institutions and home-based settings. The flexibility of the model makes it accessible to a wide range of people, regardless of their location or background. By using VR technology, people with PPPD can receive effective treatment without feeling any reluctance. This is due to the proper guidance provided by the model, which enables habituation therapy to be used to treat PPPD dizziness symptoms effectively by reducing fear and anxiety as well as promoting better health outcomes. The integration of IoT is also suggested as a way to promote contemporary technology into the developed system in order to enhance the capability of the VR experience by adding a feature.

### Limitations

Further investigation is needed to determine the applications of the V-CarE model in various settings, from medical clinics and hospitals to educational institutions and home-based environments. To ensure the feasibility of the study and the usability testing, a power analysis and sample size calculation can be performed using G*Power.

### Conclusions

In conclusion, the purpose of the V-CarE model is to provide a tool that effectively uses VR technology for interdisciplinary collaboration between health care, creative technology (in this case, VR), and education. It lays the foundation for exploring and understanding the effects of VR technology on human behavior and cognition, with its ability to address the challenges posed by the COVID-19 pandemic. Moreover, it provides effective treatment solutions for PPPD for improvements in health care outcomes, and people's lives. As the V-CarE model has the potential to make a positive impact in day-to-day activities of the users, it is, therefore, suggested to continue to conduct further research and support interdisciplinary collaboration to help bring these innovative solutions to our lives.

## Abbreviations

IoT: internet of things
PCR: polymerase chain reaction
PPPD: Persistent Postural-Perceptual Dizziness
V-CarE: Virtual Care Experience
VR: virtual reality
